# Whole‐brain 3D FLAIR at 7T using direct signal control

**DOI:** 10.1002/mrm.27149

**Published:** 2018-02-24

**Authors:** Arian Beqiri, Hans Hoogduin, Alessandro Sbrizzi, Joseph V. Hajnal, Shaihan J. Malik

**Affiliations:** ^1^ Department of Biomedical Engineering, School of Biomedical Engineering & Imaging Sciences King's College London, King's Health Partners, St Thomas' Hospital London United Kingdom; ^2^ Center for Image Sciences, University Medical Centre Utrecht Utrecht Netherlands; ^3^ Centre for the Developing Brain, School of Imaging Sciences & Biomedical Engineering, King's College London, King's Health Partners, St Thomas' Hospital London United Kingdom

**Keywords:** Direct signal control, dynamic RF shimming, FLAIR, parallel transmission, RF shimming, ultrahigh field

## Abstract

**Purpose:**

Image quality obtained for brain imaging at 7T can be hampered by inhomogeneities in the static magnetic field, B_0_, and the RF electromagnetic field, B_1_. In imaging sequences such as fluid‐attenuated inversion recovery (FLAIR), which is used to assess neurological disorders, these inhomogeneities cause spatial variations in signal that can reduce clinical efficacy. In this work, we aim to correct for signal inhomogeneities to ensure whole‐brain coverage with 3D FLAIR at 7T.

**Methods:**

The direct signal control (DSC) framework was used to optimize channel weightings applied to the 8 transmit channels used in this work on a pulse‐by‐pulse basis through the echo train in the FLAIR sequences. 3D FLAIR brain images were acquired on 5 different subjects and compared with imaging using a quadrature‐like mode of the transmit array. Precomputed “universal” DSC solutions calculated from a separate set of 5 subjects were also explored.

**Results:**

DSC consistently enabled improved imaging across all subjects, with no dropouts in signal seen over the entire brain volume, which contrasted with imaging in quadrature mode. Further, the universal DSC solutions also consistently improved imaging despite not being optimized specifically for the subject being imaged.

**Conclusion:**

3D FLAIR brain imaging at 7T is substantially improved using DSC and is able to recover regions of low signal without increasing imaging time or interecho spacing.

## 1 INTRODUCTION

Fluid‐attenuated inversion recovery (FLAIR) sequences^1^ are commonly used for assessing neurological disorders such as multiple sclerosis.^2^ Moving to higher field MRI ( ≥ 3T) enables greater SNR, which often facilitates greater image contrast.^3^ For FLAIR imaging, this has been shown to increase the number of multiple sclerosis lesions that can be observed.^4^ Ultrahigh field (UHF) ( ≥ 7T) MRI potentially offers the ability to detect even more lesions and neurological abnormalities, along with further improved SNR.

3D images can be obtained using a 3D fast spin echo (FSE) readout after a global inversion; however, this strategy becomes challenging at UHF. The substantial inhomogeneity in RF magnetic field ( $B_{1}^{+}$) produced at UHF causes significant variations in signal and image contrast, making whole brain coverage difficult. Further, the relaxation times of gray and white matter substantially increase, whereas they do not change measurably for CSF. This means that relying on T_1_ differences alone to suppress the CSF signal, while still producing suitable SNR and contrast for gray and white matter, is progressively less effective as the field strength goes up. To address this, in a study by Visser et al.,^5^ a magnetization preparation module was added to the start of the FLAIR sequence prior to the inversion pulse to increase overall signal from white and gray matter while still suppressing CSF signal. Although effective, substantial variation in signal and contrast remained, along with regions with virtually no signal due to $B_{1}^{+}$ variation. A preparation module tailored to improve SNR or image contrast when used with an optimized flip angle train was designed by Saranathan et al.^6^ and used to acquire in vivo data on multiple subjects. However, in all cases the low signal regions were still present in the images, likely due to reduced $B_{1}^{+}$.

3D FSE sequences with long echo trains typically do not use 180 ° refocusing pulses for 2 major reasons: 1) signal decay would be dictated purely by T_2_ and would hence not allow collection of very long echo trains (durations of hundreds of milliseconds typically) necessary for efficient k‐space sampling, and 2) RF power and SAR would be limiting.^3^, ^7^ To solve these problems, refocusing pulse trains with variable and lower refocusing angles have been introduced. However, as shown in reference 8, 3D FSE imaging with variable refocusing angles is sensitive to $B_{1}^{+}$ dropouts.

$B_{1}^{+}$ inhomogeneity can be reduced using parallel transmission (PTx), for which a set of independent transmit coils is used to control the RF excitation field. By changing the complex weighting applied to each channel of the array, known as *RF shimming*, the overall $B_{1}^{+}$ field can be made more uniform.^9^ Although RF shimming is able to effectively improve $B_{1}^{+}$ inhomogeneity in a single slice, it is considerably less effective over an entire volume, particularly at UHF.^10^ Its efficacy is also highly dependent on the transmit coil being used and the size of the object being imaged.

Additional degrees of freedom can be accessed by allowing RF shims to vary for each pulse in the sequence, producing spatially and temporally varying RF fields through the FSE echo train.^11^ If not properly controlled, this will violate the Carr Purcell Meiboom Gill condition, leading to a loss of stability and rapid signal loss. However, it has been demonstrated previously^11^ that the RF shims can be optimized by using a spatially resolved extended phase graph (SR‐EPG) signal model to produce spatially uniform echo amplitudes with a desired target temporal behavior: this method is referred to here as *direct signal control* (DSC). The method has previously been used for 2D imaging at 3T and 7T^11^, ^12^ and whole‐brain 3D FSE at 3T.^13^ The nonlinear optimization has been recently recast as an optimal control problem,^12^ which also allows for the enforcement of explicit peak and average power constraints. Prior work on DSC has not extended to in vivo 3D imaging at 7T and has not considered magnetization‐prepared sequences such as FLAIR.

In this work, we have used the DSC method to acquire whole‐brain 3D‐FSE FLAIR images on 5 healthy volunteers at 7T, employing strict RF power limits for safety. The feasibility of producing “universal” solutions^14^ that may be applied without subject‐specific optimization was also investigated.

## 2 METHODS

All data was acquired on a Philips 7T MRI system (Philips Healthcare, Cleveland, OH) with a Nova 8‐channel transmit and 32‐channel receive head coil (Nova Medical, Wilmington, DE). Local safety rules for working with PTx allow a maximum average power of 1 watt (W) per channel on each of the 8 channels to maintain a conservative margin for error within local specific absorption rate (SAR) limits.

The approach taken in this work was to assume that there exists an already optimized *base sequence*, which would give the desired contrast in the case that the $B_{1}^{+}$ and B_0_ fields are uniform. DSC optimization is then used to bring the true signals as close as possible to the target defined by this ideal performance. In this work, we have defined the base sequence (outlined below) based on prior work. The focus of this paper is on how the PTx degrees of freedom can be used to optimize image quality given this sequence.

### 2.1 Base sequence

**Figure 1:**
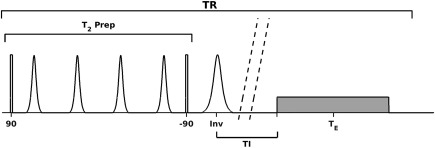
RF pulse sequence, including the preparation module, and the FSE imaging module (gray box).

The basic FLAIR pulse sequence used for this work was based on that in reference 5, in which a T_2_‐preparation module is played out prior to inversion. T_2_‐prep is used to increase the amount of available signal from brain tissue (i.e., gray and white matter) at the time of the FSE echo train, while still allowing the CSF signal to be nulled. The T_2_‐prep module used adiabatic hyperbolic secant refocusing pulses (duration = 9 ms, peak amplitude = 15 µT, maximum frequency modulation = 706 Hz) with hard 90 ° tip‐down and tip‐up pulses. The subsequent inversion was also a hyperbolic secant pulse (duration = 17.1 ms, peak amplitude = 15 µT, maximum frequency modulation = 700 Hz). Figure 1 shows the structure of the pulse sequence used.

The FSE imaging module was a vendor‐specified sequence for 3D‐FLAIR at 7T, using refocusing flip angles that quickly ramp down to a constant 50 °. The flip angle choice gives good T_2_ weighting with a low overall SAR and allows for a long echo train. The readout consisted of 192 pulses, each of 0.8 ms duration in the train, with interecho spacing 3 ms. The 3D FLAIR images were acquired with sagittal native orientation FOV = 250 × 250 × 190 mm; 1 mm isotropic resolution; TI = 2,250 ms; TR = 8 s; and acquisition time = 7.5 minutes. The long TR was required to ensure CSF has as much longitudinal magnetization as possible so that it nulls at the longest TI possible. This allows maximal recovery of signal from gray and white matter.

### 2.2 Preliminary investigation: magnetization preparation

The overall objective of this work was to correct for major signal nonuniformity seen with this sequence related to $B_{1}^{+}$ inhomogeneity at UHF. As outlined above, the sequence can be split into a magnetization preparation (MP) phase (i.e., T_2_‐prep and inversion) and a readout phase. We hypothesized that the magnetization preparation would not be the major cause of the signal nonuniformity. This was tested in a preliminary investigation by performing Bloch equation simulations over a range of relative $B_{1}^{+}$ and static magnetic field (B_0_) nonuniformity. Prior work by Visser et al.^5^ used a T_2_‐prep module with 4 adiabatic refocusing pulses, whereas work from Saranathan et al.^6^ used a similar sequence with 2 refocusing pulses. Both variants were simulated here.

For each $B_{1}^{+}$ and B_0_ combination, single isochromat Bloch simulations were used to assess the effect of the preparation module, combined with EPG simulations of the FSE readout block. Perfect spoiling of transverse magnetization between preparation and readout modules was assumed. The simulation was repeated over multiple repetition times to ensure a steady state was achieved. Convergence generally occurred after 3 periods. For simplicity, the simulations were performed for CSF with T_1_ = 4,300 ms and T_2_ = 2,000 ms; and “average brain tissue” with T_1_ = 1,500 ms and T_2_ = 50 ms.^15^

The results of the preliminary investigation will be presented later; however, the overall conclusion was that it would be sufficient to transmit the MP module in the quadrature mode of the RF transmit coil and to reserve the PTx degrees of freedom for the 3D‐FSE readout only. Furthermore, a T_2_‐prep module with 4 refocusing pulses was preferred to the 2‐pulse version. Full details are in the Results section.

### 2.3 PTx and DSC optimization

Based on the working hypothesis that the MP module was relatively insensitive to B_0_/ $B_{1}^{+}$, the parallel transmit degrees of freedom were used only for a DSC optimization of the 3D‐FSE readout. As previously stated, the DSC optimization aims to minimize the difference between the predicted echo amplitudes in the subject (as a function of space and echo number) and a target signal. Within this framework, both the predicted and target echo amplitudes are defined with respect to some reference T_1_ and T_2_ times, which in this work were set to be approximately indicative of average brain tissue at 7T; $T_{1}^{\text{ref}}=1,500$ ms and $T_{2}^{\text{ref}}=50$ ms. The predicted echo amplitude for the *i^th^* spatial location and *j^th^* echo $I_{ij}=I\left(r_{i},j\right)$ is then computed by using the SR‐EPG model^11^ $I=f\left(w,S,a,T_{1}^{ref},T_{2}^{ref}\right)$, in which $w_{jk}$ is the complex weighting value (i.e., RF shim) applied to the *j* ^*th*^ RF pulse for the *k* ^*th*^ channel; $S_{ik}=S_{k}\left(r_{i}\right)$ is the spatial sensitivity of the *k* ^*th*^ channel at the *i* ^*th*^ spatial location (*r_i_*); and $a_{j}$ is the flip angle of *j* ^*th*^ pulse in the base sequence. In this notation, the spatial sensitivity $S_{k}\left(r_{i}\right)$ is a dimensionless quantity that specifies the achieved $B_{1}^{+}$ to a desired reference value, obtained via $B_{1}^{+}$ mapping. RF shim parameters *w*_*jk*_ are also dimensionless (but complex) scaling factors, and the RF coil is calibrated such that setting equal amplitude to each channel (i.e., ***w*** = [1 1 1 1 1 1 1 1]) results in nominal quadrature excitation. Note that B_0_ inhomogeneity is not part of the SR‐EPG model because the RF pulses are short ( < 1 ms), meaning they have large bandwidths compared with observed field nonuniformity. The target signal $T_{ij}=T\left(r_{i},j\right)$ is the behavior of the sequence under ideal conditions for a single‐channel transmitter with uniform spatial sensitivity, that is, $T=f\left(1,1,a,T_{1}^{ref},T_{2}^{ref}\right)$. In this case, *T*_*ij*_ is constant for all space (index *i*).

The DSC optimization is then formulated according to Equation (1):

(1) $${\text{min}}_{w_{jk}}\sum_{i,j}{\left\|C_{ij}\circ \left(I_{ij}-T_{ij}\right)\right\|}_{2}^{2}$$

$$\text{such that}\;\underset{j=1:N_{p}}{\sum }{\left|w_{jk}b_{j}\right|}^{2}\frac{t_{RMS,j}}{TR}A\leq P_{avg}\;\forall \;k=1:N_{c}$$

$${\left|w_{jk}b_{j}\right|}^{2}A\leq P_{peak}\;\forall \;j=1:N_{p},k=1:N_{c},$$

where *i,j,k* are indices over space, pulses, and transmit channels, respectively; *N_p_* is the number of RF pulses in the echo train; *N_c_* is the number of transmit channels; and *C_ij_* is a weighting function ( ∘ denotes element‐wise multiplication of these matrices). Constraints are written in terms of the RF pulse properties: for a pulse shape *p(t), b* is the peak B_1_ in units of µT and $t_{RMS}=\int^{}p^{2}\left(t\right)dt/b^{2}$ is a constant that relates to RF power. Both quantities can be variable throughout the echo train if different pulse shapes are employed (e.g., typically different shapes are used for excitation and refocusing). *A* is a coil‐ and amplifier‐dependent power conversion factor that may typically be measured by using an RF power meter and normalizing to the reference $B_{1}^{+}$. In this work, *A* = 0.35 W/µT^2^. The peak and average power limits are denoted by *P*_*peak*_ and *P*_*avg*_ and were set to 85W per channel and 1W per channel, respectively.

**Figure 2:**
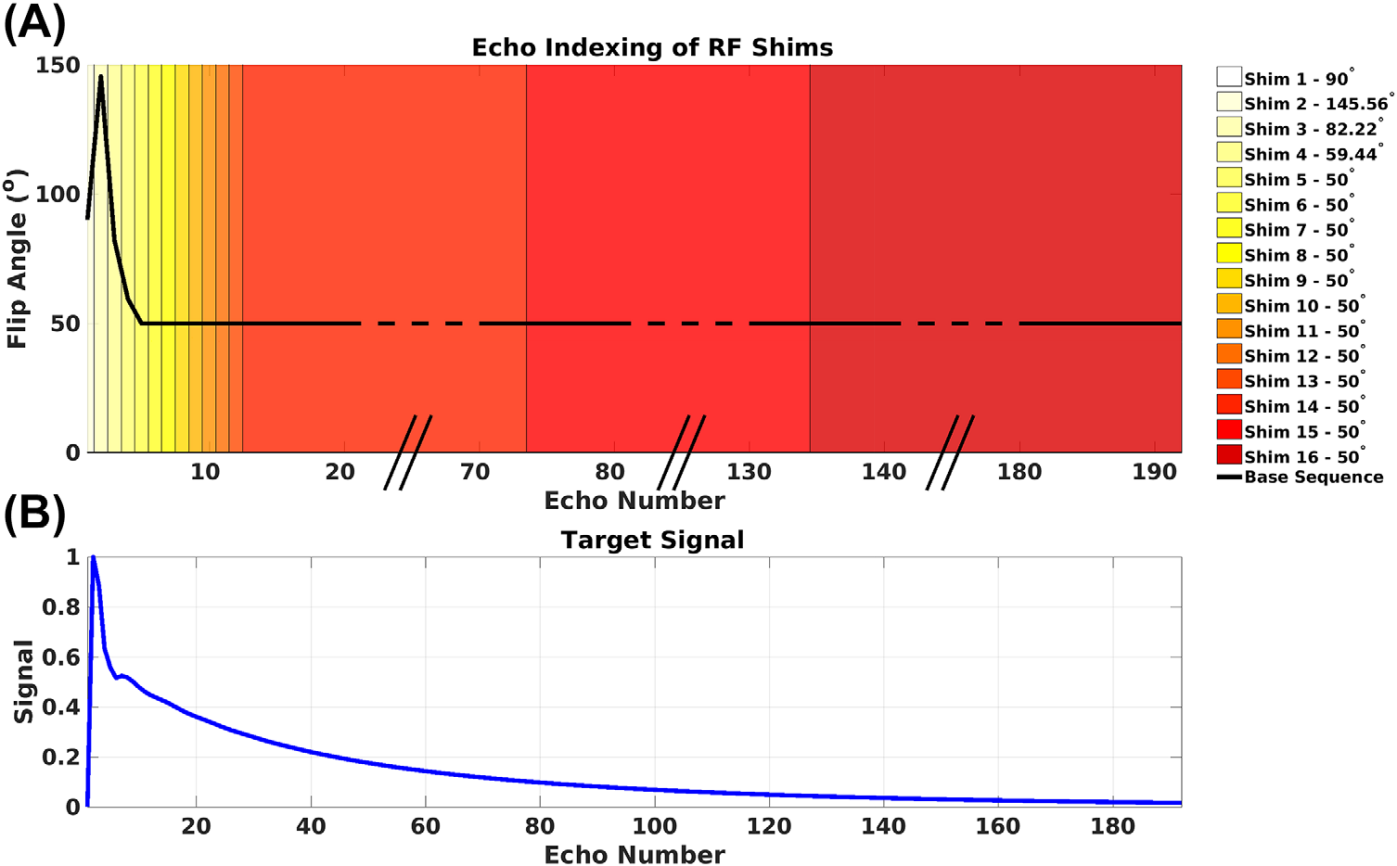
(a) Flip angles in the base sequence are shown with the black line. The shims applied to each RF pulse are shown by the heat map in the background of the figure. (b) Target signal (***T***) is the same for all spatial locations.

The DSC algorithm can in principle compute separate optimized RF shim settings (for the 8‐channel transmit coil) for each of the 192 pulses in the echo train; however, the cost is that each element of ***w*** (192 × 8 complex matrix) must be computed. In previous work, it was shown^12^, ^13^ that good results may be achieved by reducing the number of degrees of freedom by applying a mapping $w'=P^{-1}w$, for which ***w′*** is a matrix of size $N_{s}\times N_{c}$; $N_{s}$ is a smaller number of independent RF shim settings; and ***P*** is a mapping matrix. In this work, we set *N_s_* = 16 and mapped the first 13 rows of ***w*** to the first 13 rows in ***w′***, whereas the remaining rows of ***w*** are mapped in blocks of 60, 60, and 59 to the last 3 rows in ***w′***. The optimization is reduced to computing the elements of ***w′***; the allocation of RF shims to the pulses is shown in Figure 2.

The optimization was performed using the optimal control formalism described in reference 12 using the interior point algorithm of the fmincon function in MATLAB (The Mathworks, Natick, MA, USA). Because the sequence TR was 8 s, steady‐state effects^13^ were not considered for the reference tissue; CSF would normally need such consideration but is nulled within the FLAIR sequence. Weighting function *C_ij_* was spatially uniform but ramped up and down linearly through the echo train to increase the relative importance (and hence spatial homogeneity) of the center of *k*‐space echoes, with maximum weighting given to the central echo. Optimizations were initialized with all elements of ***w′*** set to be 0.6 (i.e., quadrature mode but with reduced amplitude) to avoid violating constraints early in the calculation.

Calculations were performed online on a Lenovo ThinkPad x230 laptop (Lenovo Group Ltd, Morrisville, NC) with an Intel i5 single‐core processor (Intel Corp., Santa Clara, CA) and required approximately 5 minutes to complete on average. An open‐source repository for the software used in this work is available at https://github.com/mriphysics/optimal-control-EPG.

### 2.4 Imaging experiments and “universal” solution

Ten subjects were scanned in total, with all participants providing written informed consent prior to enrollment. For the first 5 subjects, $B_{1}^{+}$ and B_0_ maps were acquired (details below). These 5 subjects were used for optimization of the MP module; data for the 2‐refocusing pulse T_2_‐prep module was acquired on 3 of these subjects. The $B_{1}^{+}$ maps from all 5 initial subjects were used to compute a “universal” DSC solution^14^ by performing the same optimization as outlined in Equation (1) but with the transmit sensitivity maps $S_{k}\left(r_{i}\right)$ for each subject concatenated together to form a large matrix. Essentially, this universal DSC solution minimized the least‐squares error over the whole test group. The advantage of a universal solution would be to obviate the need for online optimization and acquisition of $B_{1}^{+}$ maps.

The remaining 5 subjects out of the 10 who were scanned were then imaged for the FLAIR experiments. For these, $B_{1}^{+}$ and B_0_ maps were acquired to compute individually optimized DSC solutions. B_0_ shimming was performed using an image‐based method.^16^ 3D $B_{1}^{+}$ mapping was performed by combining a quantitative quadrature mode acquisition using the dual refocusing echo acquisition mode technique,^17^, ^18^ with a series of per‐channel low flip angle gradient echo images.^19^ These were all acquired with 3.5 mm isotropic resolution. The dual refocusing echo acquisition mode acquisition (flip angle = 7 °, STEAM angle = 40 °) took 16 s, and the gradient echo scans took 165 s to acquire all transmit channels.

3D‐FLAIR images were acquired using the outlined DSC approach, with both individually optimized and universal solutions for all 5 subjects. Matched protocols using the quadrature mode of the RF coil and static RF‐shimming were also acquired for comparison. Static RF shimming was performed using a constrained magnitude least squares type optimization^20^ based on the variable exchange method,^21^ utilizing the CVX convex optimization package to constrain peak and average power.^22^, ^23^ RF shim and DSC optimizations were all performed over whole‐brain 3D masks, which were automatically created using the FSL (functional MRI of the brain [FMRIB] software library) brain extraction tool.^24^

### 2.5 Image postprocessing and analysis

To analyze the contrast variation in the acquired images, the data was postprocessed using the FSL FMRIB Automated Segmentation Tool (FAST) bias‐correction tool^25^ and registered using FSL FMRIB Linear Image Registration Tool (FLIRT).^26^, ^27^ FAST jointly estimates bias field and image segmentation using a Gaussian mixture model. We used the image segmentation quality as an indicator of the underlying contrast uniformity.

## 3 RESULTS

### 3.1 Magnetization preparation preliminary investigation

**Figure 3:**
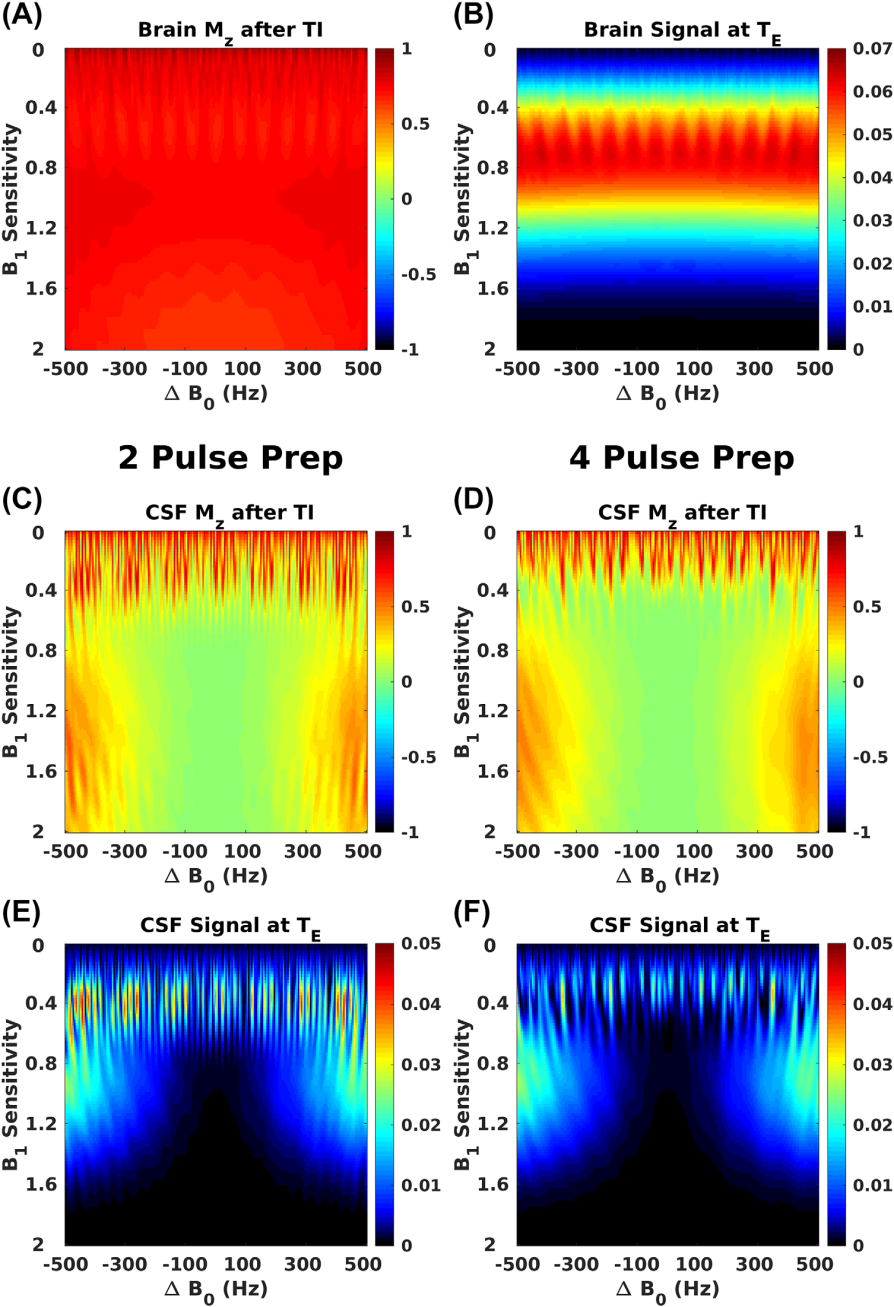
(a) Simulated longitudinal magnetization of brain tissue for a range of B_1_ sensitivities (*S*(***r***) in our notation) and B_0_ off‐resonances at the start of the imaging module using the full 4‐pulse preparation module; and (b) brain signal computed from SR‐EPG calculations at the center of the echo train. (d) and (f) show the same for CSF for the full preparation module, and (c) and (e) show the data for CSF for the 2‐pulse preparation module. SR‐EPG, spatially resolved extended phase graph.

Figure 3a shows the simulated longitudinal magnetization for brain tissue at the start of the imaging module. For most of the $B_{1}^{+}$ and B_0_ values, it is clear that the longitudinal magnetization is relatively homogeneous and has recovered substantially after the delay TI. The resultant signal from brain tissue at the center of the echo train (simulated via EPG calculation) is shown in Figure 3b, and it is clear that variation due to B_1_ inhomogeneity is considerably greater than that caused by the magnetization preparation module.

**Figure 4:**
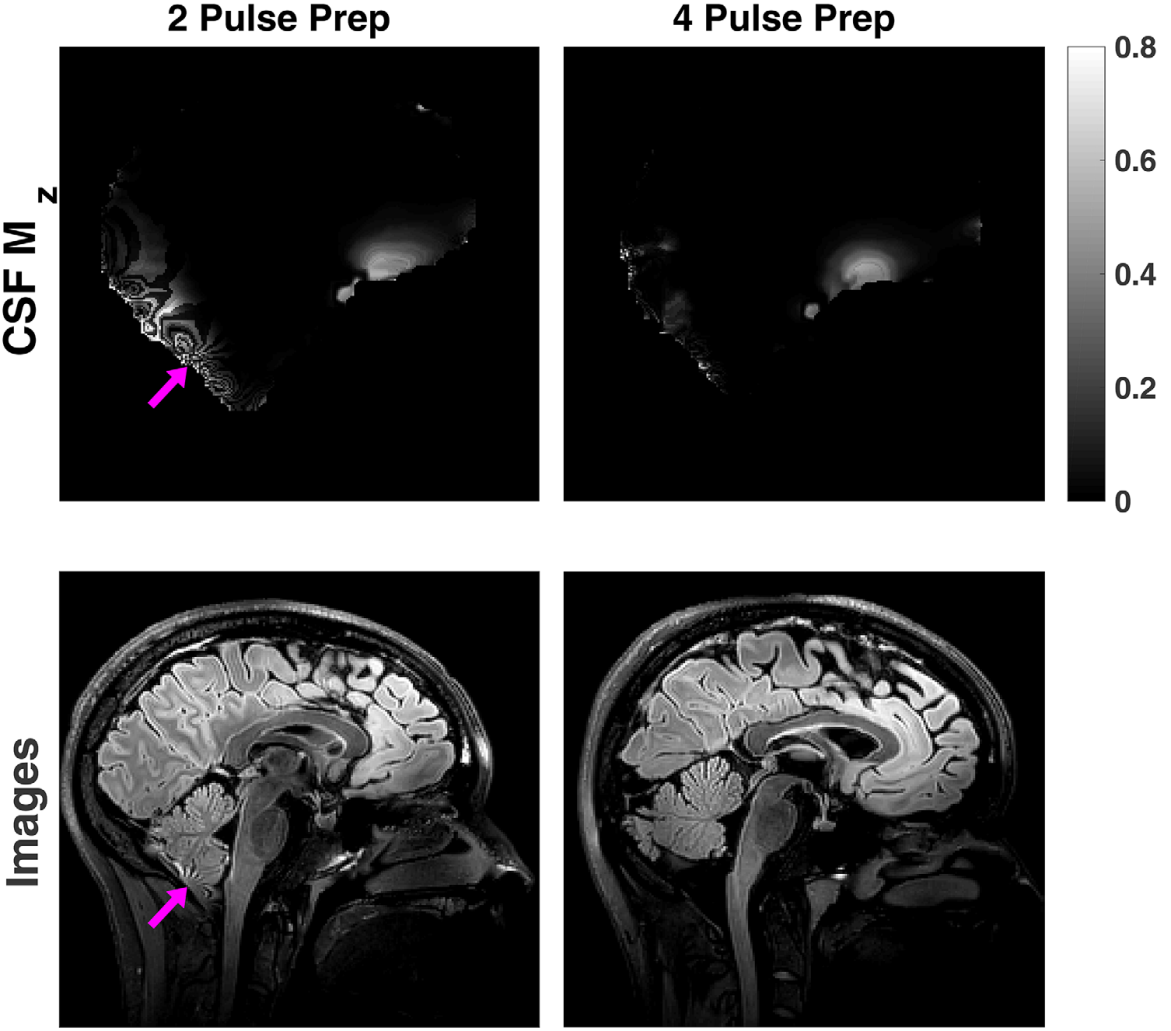
Top row: Simulated longitudinal magnetization in CSF at time TI when using 2‐refocusing (left) and 4‐refocusing (right) MP modules. Data are from 2 different volunteers. Corresponding B_1_ sensitivity and B_0_ field data are shown in Supporting Information Figure S1. Bottom row: 3D‐FLAIR images obtained for each subject. The fringe‐like artefact visible in the 2‐refocusing images (arrow) is predicted qualitatively by the simulations. Such artefacts were observed in all volunteer datasets acquired using the 2‐refocusing MP module. FLAIR, fluid‐attenuated inversion recovery; MP, magnetization preparation.

Figures 3d and 3f show the same data for CSF for the 4 refocusing pulse T_2_‐prep module. Similar simulations with the 2 refocusing pulse version are shown in Figures 3c and 3e. It is clear that the latter is more sensitive to off‐resonance in regions where the $B_{1}^{+}$ sensitivity is low. Figure 4 shows examples of 3D‐FLAIR images obtained using both sequences in quadrature mode, compared with predicted M_z_ in CSF at the start of the FSE readout, which is formed by mapping the results of Figure 3 onto measured $B_{1}^{+}$ and B_0_ field data for these subjects (field data is shown in Supporting Information Figure S1). The simulated predictions qualitatively agree well with the observed images, suggesting that “fringe‐like” artefacts observed in the images obtained from the 2‐refocusing pulse preparation could be attributed to B_0_ sensitivity of the 2‐pulse MP module at low $B_{1}^{+}$. Similar artefacts were seen in all FLAIR images acquired using the 2‐refocusing pulse MP and were not present in acquisitions with the 4‐refocusing pulse MP module.

The conclusion of this preliminary investigation was to support the initial hypothesis that the predominant cause of the signal nonuniformity in 3D FLAIR imaging is the 3D FSE readout, with the MP module having a very small contribution. The MP module, however, is sensitive to B_0_ inhomogeneity at low $B_{1}^{+}$, particularly when using only 2 refocusing pulses. Hence, the 4‐refocusing pulse T_2_‐prep module was adopted using quadrature excitation, and DSC optimization focused solely on the 3D‐FSE readout. The cost of this was that the MP module contributed 0.34W of average RF power (limit 1W) compared with 0.15W for the 2‐pulse preparation module. The average power contribution of the MP module was accounted for in the DSC optimization.

### 3.2 3D FLAIR imaging data

**Figure 5:**
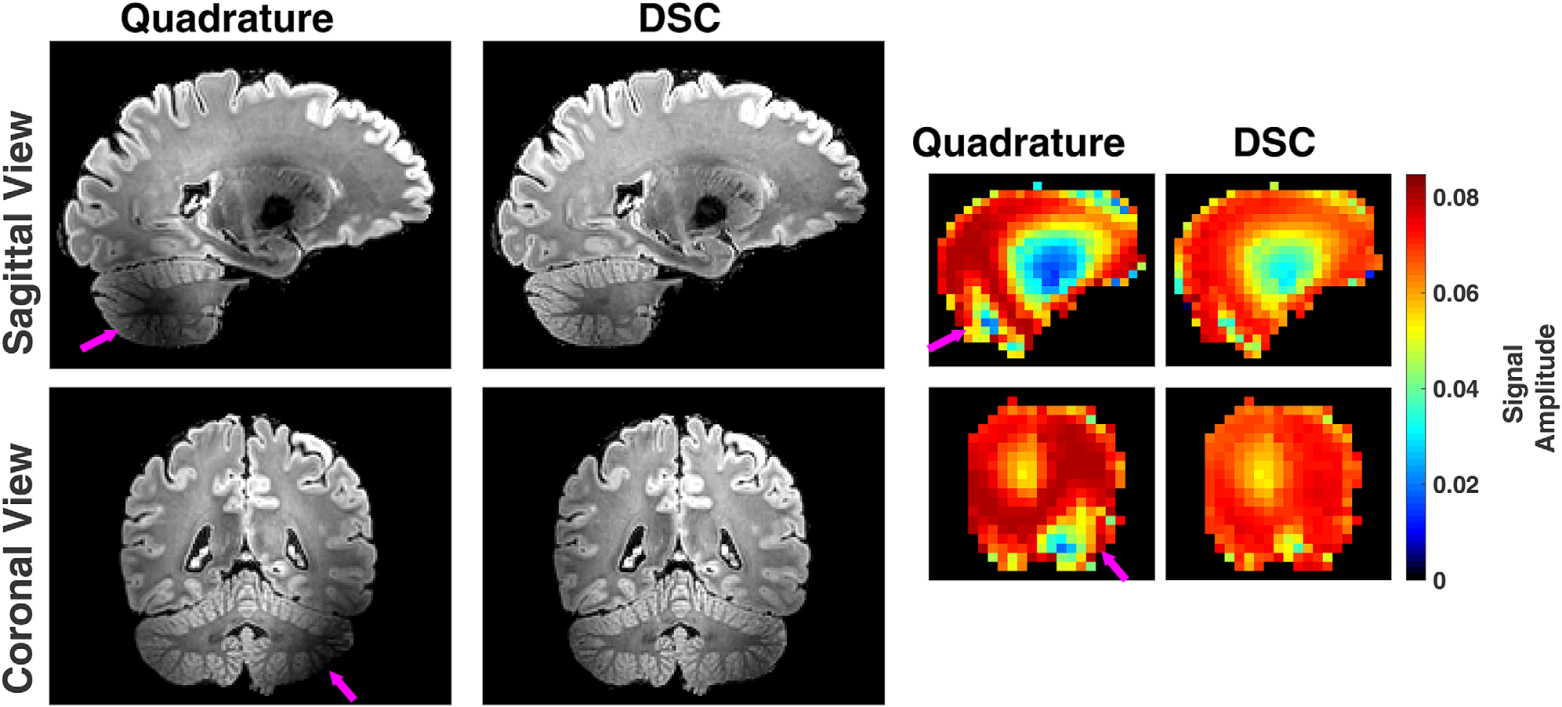
Acquired images are shown on the left for quadrature and DSC in both sagittal and coronal views in slices through the cerebellum. Regions of low signal in the cerebellum and center of brain are effectively recovered with DSC, as predicted by the corresponding SR‐EPG simulation data shown on the right. DSC, direct signal control.

Figure 5 compares 3D‐FLAIR images obtained using quadrature mode excitation with DSC. DSC was able to recover regions with low signal, ensuring whole brain coverage. The imaging data on the left side of Figure 5 shows one example with increased signal in the cerebellum and center of the brain. The right side of Figure 5b shows the SR‐EPG model predictions for the center of k‐space echo. The predictions match the signal intensity patterns seen in the acquired data.

**Figure 6:**
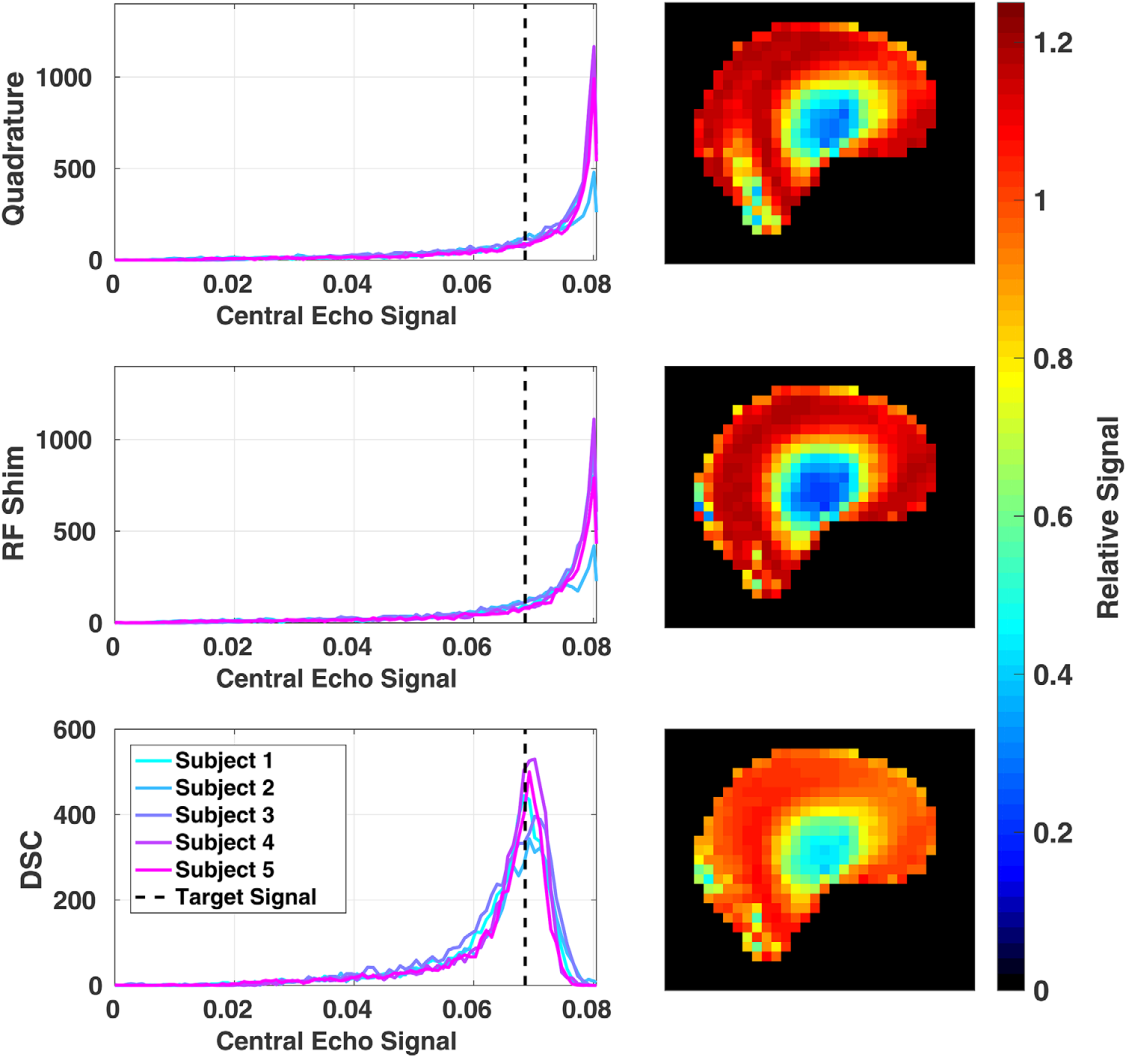
Histograms of the central echo prediction from EPG calculations for all subjects over the whole brain volume, with the target signal shown with a dotted line. On the right, the predicted signal relative to the target echo signal in a single slice is shown for a subject in all 3 cases.

As Figure 5 demonstrates, the image quality does follow the SR‐EPG model predictions quite well. Figure 6 shows whole‐brain image histograms computed from the SR‐EPG echo amplitude prediction for the 100th echo (center of k‐space). Figure 6 also shows a single slice from the prediction for one volunteer for each method. The DSC optimization leads to the most spatially uniform signal.

**Figure 7:**
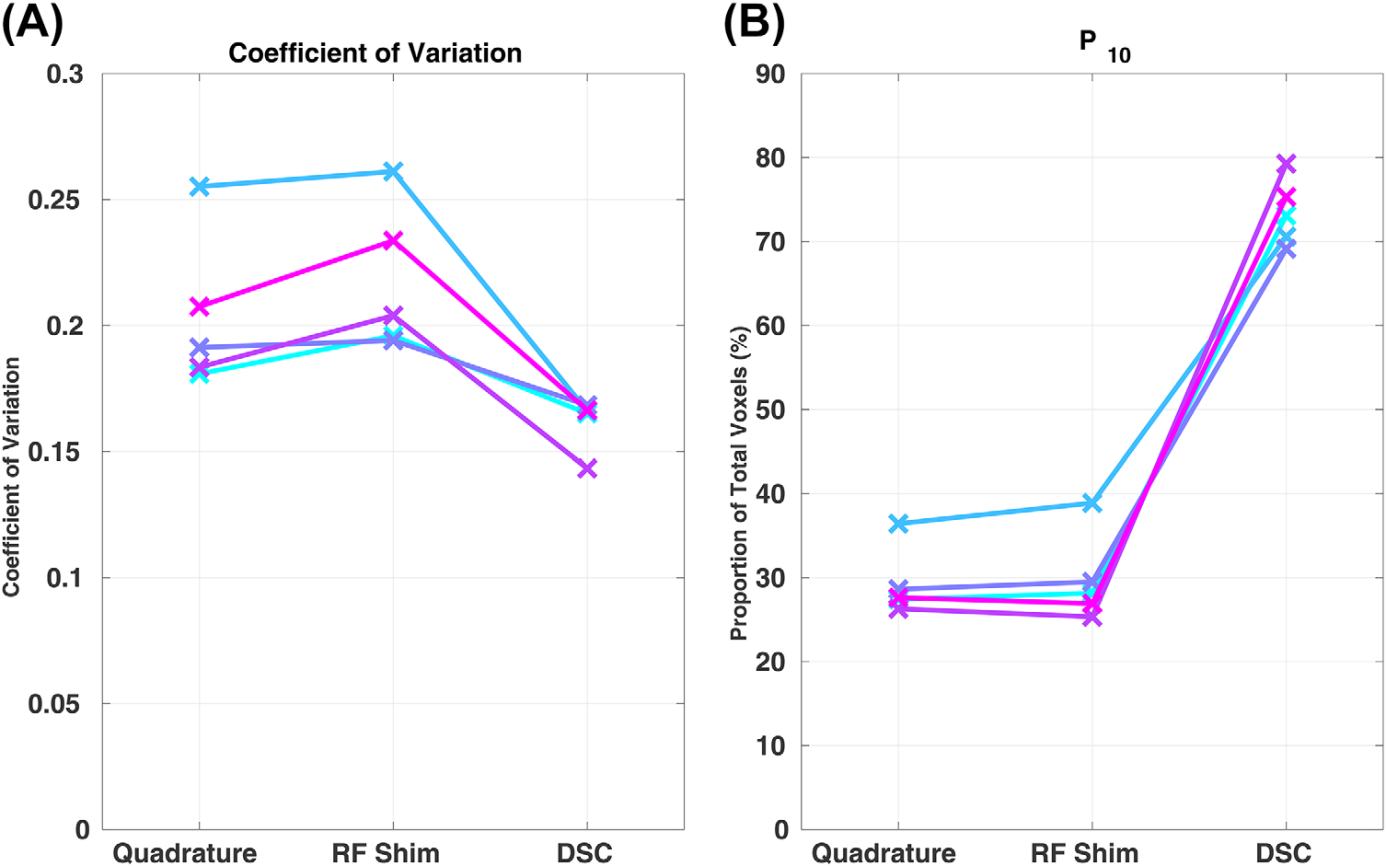
Coefficient of variation (a) and P_10_ (proportion of voxels that fall within 10% of the target signal) (b) in the central k‐space echo over the whole brain volume for all the subjects for quadrature, RF shimming, and DSC.

Figure 7 shows the coefficient of variation of the predicted central k‐space echo, along with the proportion of voxels that fall within 10% of the target signal (P_10_). The latter measure was introduced because the histograms in Figure 6 are clearly non‐Gaussian; hence, coefficient of variation has limited meaning here. It can be observed that DSC always substantially improves the homogeneity of signal (reduces coefficient of variation, increases P_10_), whereas RF shimming has a limited effect — and in many cases slightly worsens it.

**Figure 8:**
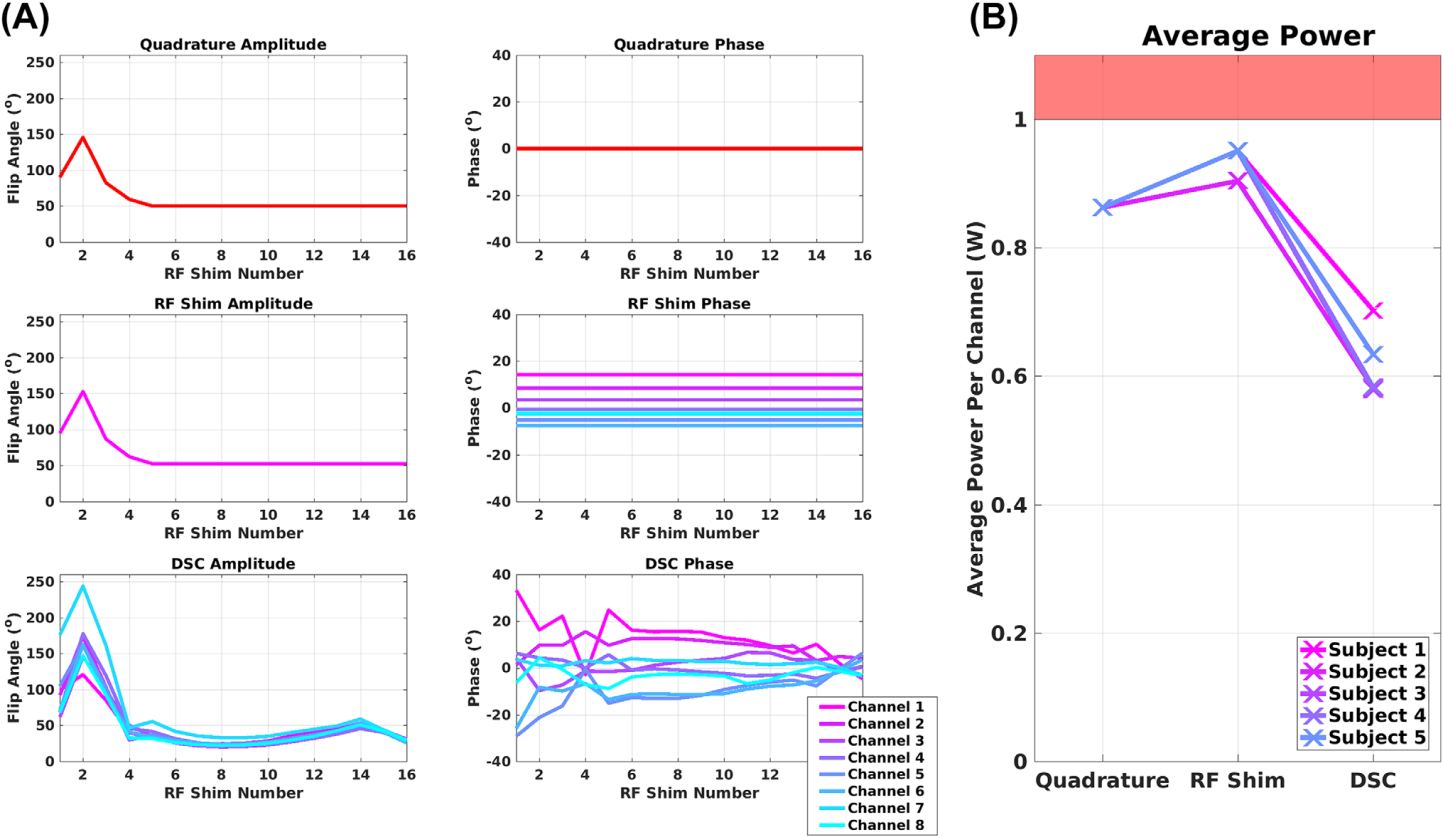
(a) Optimized RF shims for a single subject. The amplitude and phase plotted here is the product of the base flip angle *a_j_* with the RF shim setting *w_jk_*. This shows the variation in *nominal* flip angle and phase per channel over the pulse sequence. The index RF shim number corresponds to the different dynamically switched RF shim settings (*w_jk_*) for the DSC case. For the quadrature and static shim cases, these are not switched. Only the flip angle *a_j_* is changing. The quadrature mode plot is simply the base sequence for indicative points in the echo train. (The total number of echoes was 192, not 16.) In the DSC case, the amplitude and phase applied from each RF channel is differentially modulated over the course of the echo train. This is the degree of freedom that the DSC method seeks to exploit. (b) Average power per channel (average over channels) shown for all 5 subjects.

Figure 8a shows the flip angle trains used for 1 volunteer. RF shimming and DSC solutions are illustrated by multiplying the base sequence flip angles by the RF shim amplitude and phase for each channel; hence, each channel has a separate flip angle. RF shimming did not change the relative amplitude of each channel. This was because the sequence was heavily limited by average power; hence, each channel was driven as strongly as possible. Instead, the RF shim solution altered the relative phase of each channel in a way that remains constant through the sequence. The DSC approach introduces strong modulation in both amplitude and phase throughout the echo train, illustrating the degrees of freedom on which DSC relies to create better signal uniformity. Fig 8b shows that the DSC solution also results in an overall reduction in average RF power, in part because the flip angles of the later refocusing pulses are reduced in this method.

**Figure 9:**
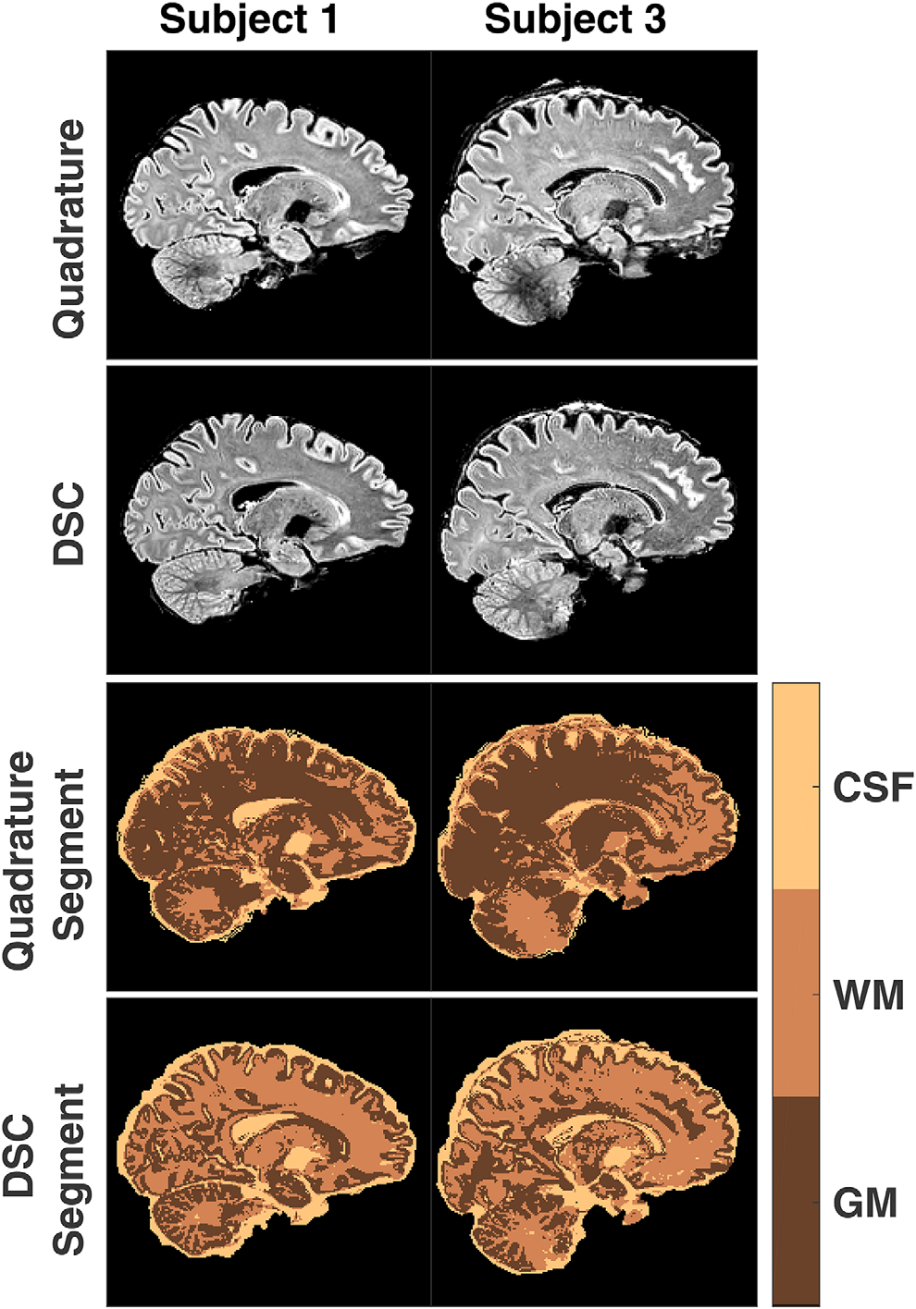
Images after bias correction for quadrature and DSC for subjects 1 and 3 are shown at the top (the data for the remaining subjects is shown in Supporting Information Figure S2). Their corresponding segmentation data into 3 tissue classes are shown below.

Figure 9 shows quadrature and DSC images after bias field correction for 2 subjects. It can be observed that the noise in regions with low signal in the images acquired in quadrature mode is greater than in the images acquired with the DSC pulses. Figure 9 also shows image segmentation results. A 3‐class mixture model was used. These classes can be broadly identified as gray matter, white matter, and CSF. This is particularly true for DSC data compared with quadrature acquisition, indicating that the 3‐class model fits the DSC data more consistently with the known anatomy. However, the segmentation is not perfect. Deep gray nuclei are classified in a single group with CSF. Supporting Information Figure S2 has equivalent data for the other 3 subjects.

**Figure 10:**
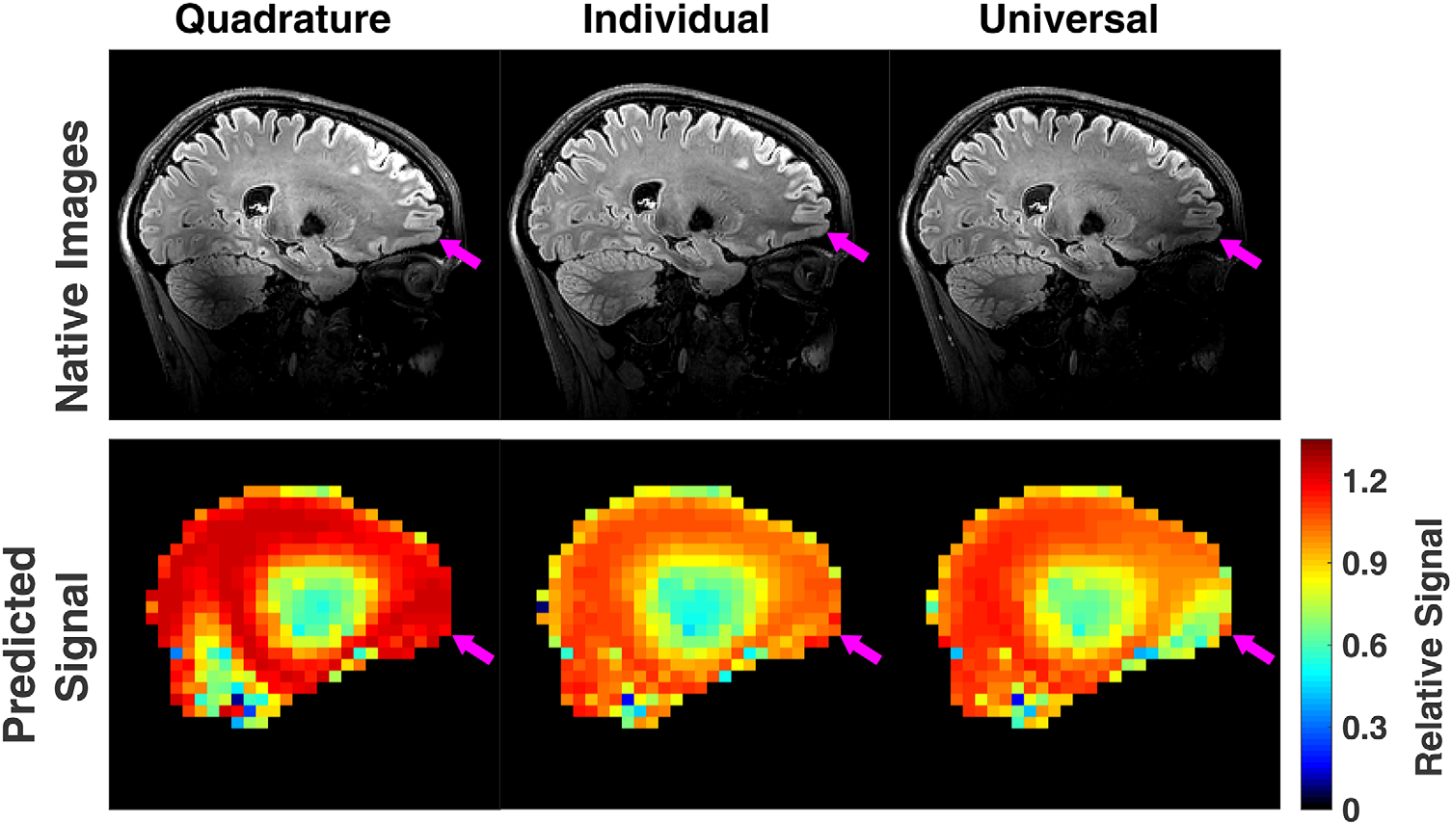
Images and predicted central k‐space signal relative to the target signal for quadrature, individual DSC, and universal DSC. Arrows highlight a region of low signal in frontal region produced when using the universal DSC solution.

Figure 10 shows an example dataset obtained when using the universal DSC solution for 1 subject. The universal solution is able to improve the image quality compared with quadrature, particularly in the cerebellum. However, in this case it is not quite as effective as the individual DSC solution. This is particularly evident at the front of the brain, where the universal solution produces some signal loss. Supporting Information Figure S3 compares the individual and universal DSC solutions for all 5 subjects. Supporting Information Figure S4 shows the coefficient of variation and P_10_ value in predicted signal for the central k‐space echo over the whole brain for all subjects for both individual DSC and universal solutions. It can be observed that the performance of the universal DSC is still effective but more variable.

## 4 DISCUSSION

In this study, whole brain 3D FLAIR imaging with no low signal regions was shown to be feasible at 7T using parallel transmission with DSC optimization. Previous studies on 3D FLAIR at 7T showed signal dropouts even after sequences had been optimized with the addition of a T_2_‐preparation module^5^ and an optimized FSE flip angle train.^6^ The DSC method has been proposed previously^11^, ^12^, ^13^ as a means for optimizing FSE sequences using PTx by modulating RF shim settings through an echo train. The method works by using a spatially resolved EPG calculation for a specific reference T_1_, and T_2_ values as a forward model, which is then used to optimize the RF shim settings throughout a multi‐echo sequence to improve the predicted echo amplitudes with respect to some predefined target. In this work, we have extended prior work on methods development to perform a first study on multiple in vivo subjects at 7T, directly optimizing within strictly defined RF power limits imposed by the institute. Further, we have shown for the 3D FLAIR case that the major determinant of the image quality is the 3D‐FSE readout rather than the MP; thus, the former may be optimized independently. It was also found that T_2_‐preparation using 2 refocusing pulses could lead to fringe artefacts that are predicted to some degree by simulation; thus, a more costly (in terms of RF power) 4‐refocusing pulse version was used instead. In contrast with prior work, which used long T_2_ (i.e., fluid) components as reference tissues^13^ because these dominate normal T_2_‐weighted scans, this work considered shorter T_2_ tissue components as a reference because the fluid components are nulled in FLAIR imaging. The result is a simplification of the method in that a steady state over multiple TR periods need not be considered.

Results suggest that individually optimized DSC outperforms quadrature or *static* RF shimming (i.e., not modulated over the echo train), resulting in greater signal homogeneity over the entire brain volume in all subjects tested. Note that static RF shimming did not perform significantly better than quadrature excitation (Figures 6 and 7) when calculated over the whole brain. Our implementation of magnitude least squares shimming could potentially have been improved by using a more complex numerical method^28^; however, we do not believe this would lead to significantly improved results because the main issue was the highly constraining RF power limits, meaning that in effect only the phase of each channel was modified by the optimization. The DSC optimization has more freedom because it can adjust the balance of RF power through the echo train: Figure 8 shows that DSC led uniformly to a decrease in average RF power; Figure 5 shows an example of the improved resulting image quality; and Figure 7 shows quality metrics computed from the SR‐EPG forward model for the whole subject group. DSC leads to improved, and also much less variable, image quality than the alternatives. The improvement in image quality was echoed by the results of bias‐field correction/image segmentation using FSL‐FAST (Figure 9) (Supporting Information Figure S2), which produced more consistent results when starting with the DSC images. This indicates a better uniformity of contrast in these images, closer to the 3‐class tissue model used by the FAST algorithm.

A preliminary feasibility study of a universal DSC approach showed some promise (Figure 10) (Supporting Information Figure S4), with the approach consistently outperforming quadrature (and RF shimmed) excitations. As might be expected, results were found to be more variable than individually optimized DSC. Nevertheless, a universal approach would clearly be an attractive alternative to online calculation and would also remove the need to acquire calibration $B_{1}^{+}$ maps. The preliminary study presented in this work provides evidence that a universal approach to DSC is viable; however, a larger and more detailed study using a much larger training dataset is necessary future work.

The DSC method aims to optimize the predicted signal with respect to a predefined target; choice of this target is therefore important. In this work, we have adopted the philosophy that a predefined base sequence can be defined that has optimal contrast; our method then aims to reproduce this. As can be seen from Figure 3, the resulting DSC images are predicted to have a more uniform signal; however, quadrature excitation will lead to a higher overall signal in some regions of the brain. In terms of the problem as defined, this higher signal is not optimal because it differs from the target; however, it would be possible to reformulate the optimization to maximize signal should this be beneficial. Another possibility might be to simultaneously optimize for a number of different reference relaxation times and to aim for spatial uniformity of contrast between them. It would also be possible to have spatially nonuniform targets or to apply a weighting function (*C_ij_*) that is spatially nonuniform to prioritize signal behavior in certain regions. An optimization of the base sequence depends more specifically on the clinical application. The one used in this work provides a general T_2_‐weighted contrast at low RF power; however, others have been specifically optimized for particular tissue contrast or lesion detection, for example.^6^ This sort of sequence, with swept flip angles,^29^ could also be adopted into the DSC framework, but the number of independent RF shims used (*N_s_*) potentially may need to be adjusted for best results.

As stated previously, the conclusion of a preliminary investigation was that the MP module could be excluded from the DSC optimization because Figure 3 suggested that the largest cause of inhomogeneity comes from the 3D‐FSE readout. A more sophisticated approach that also optimizes the preparation module, either jointly or perhaps separately, could lead to enhanced performance by reducing the RF power required for MP (the adiabatic pulses require high RF power), leaving more overhead for the FSE readout. Attempts to reduce the power of the prep module by using only 2 adiabatic pulses led to artefacts in the CSF signal, as shown in Figure 4. However, in reference 6 an optimized preparation module with just 2 adiabatic refocusing pulses was used. Although similar fringe artefacts were seen in some cases (attributed to CSF flow in the paper), these disappeared when using a different FSE flip angle sweep. Thus a lower power, 2 refocusing pulse preparation module could potentially be used in some cases.

An alternative to the DSC approach taken in this work is to replace the individual refocusing pulses with homogeneity‐correcting PTx pulses. This was performed using kT‐points pulses in reference 30 and kT‐points pulses designed with a phase‐free rotation using the gradient ascent pulse engineering algorithm in reference 31. The tradeoff in this case is that the resulting RF pulses are longer, leading to a longer interecho spacing (9 ms in Massire et al.^31^ versus the 3 ms in this work), and thus reducing the number of echoes that can be collected for a given echo time. This approach also requires more RF power. The DSC approach utilizes only the very short pulses in a standard FSE sequence, instead exploiting the degrees of freedom of interaction over the whole pulse train to improve the signal. In the future, some combination of DSC and PTx pulses might be possible, in which a short PTx pulse is optimized using DSC instead of a pulse design that focuses on individual RF pulses.

Further overhead to improve the DSC solutions could be provided by incorporating SAR limits and an appropriate corresponding SAR model into the optimization. This would allow the 1W average power limit to be relaxed, which could lead to performance improvement because this limit is considerably more constraining than the relevant SAR limits. Optimal control DSC has recently been extended to include this by Sbrizzi et al.^32^; therefore, the SAR‐constrained framework could be used in the future.

## 5 CONCLUSION

DSC dynamic RF shimming has been used to substantially improve image quality for 3D FLAIR imaging at 7T without requiring increased echo times. The method consistently outperforms quadrature excitation and RF shimming with an 8‐channel PTx head coil and leads to consistent image quality across a group of 5 subjects, with much lower variability than quadrature excitation. A preliminary investigation into identification of a universal solution that could be applied to any subject showed some promise, but further work is needed to characterize this over a wider test group.

## Supporting information

Additional supporting information may be found in the online version of this article.

**FIGURE S1** $B_{1}^{+}$ maps (top row) and B_0_ maps (bottom row) used to produce the predictions in Figure 4. Data are from two separate subjects: the subject whose FLAIR imaging was performed with the 2‐refocussing pulse MP is on the left and the 4‐refocussing pulse MP subject is on the right.
**FIGURE S2** Bias corrected and segmented images shown as in Figure 9 for the remaining three subjects.
**FIGURE S3** a) Coefficient of variation in simulated signal for the central k‐space echo over the whole brain shown for individual DSC (“DSC”) and universal DSC (“universal”) solutions. b) The P_10_ value is shown for the same datasets. The universal solutions lead to a larger spread in these metrics than the individually optimised solutions, however all are lower than the quadrature mode and static shimming cases (Fig. 7).
**FIGURE S4** Example image slices through the cerebellum in a) coronal view and b) sagittal view for all 5 imaged subjects for quadrature, individual DSC (“DSC”) and universal DSC (“universal”) solutions.
