# Supplementary material for: Whole‐brain 3D FLAIR at 7T using direct signal control
Source: Magn Reson Med. 2018 Feb 24;80(4):1533–45. doi: 10.1002/mrm.27149 (PMC6120540; doi:10.1002/mrm.27149)
Supplement: Supplementary file 1 — FIGURE S1 B1+ maps (top row) and B0 maps (bottom row) used to produce the predictions in Figure 4. Data are from two separate subjects: the subject whose FLAIR imaging was performed with the 2‐refocussing pulse MP is on the left and the 4‐refocussing pulse MP subject is on the right. FIGURE S2 Bias corrected and segmented images shown as in Figure 9 for the remaining three subjects. FIGURE S3 a) Coefficient of variation in simulated signal for the central k‐space echo over the whole brain shown for individual DSC (“DSC”) and universal DSC (“universal”) solutions. b) The P10 value is shown for the same datasets. The universal solutions lead to a larger spread in these metrics than the individually optimised solutions, however all are lower than the quadrature mode and static shimming cases (Fig. 7). FIGURE S4 Example image slices through the cerebellum in a) coronal view and b) sagittal view for all 5 imaged subjects for quadrature, individual DSC (“DSC”) and universal DSC (“universal”) solutions. [file MRM-80-1533-s001.docx]

**Supporting Figures**


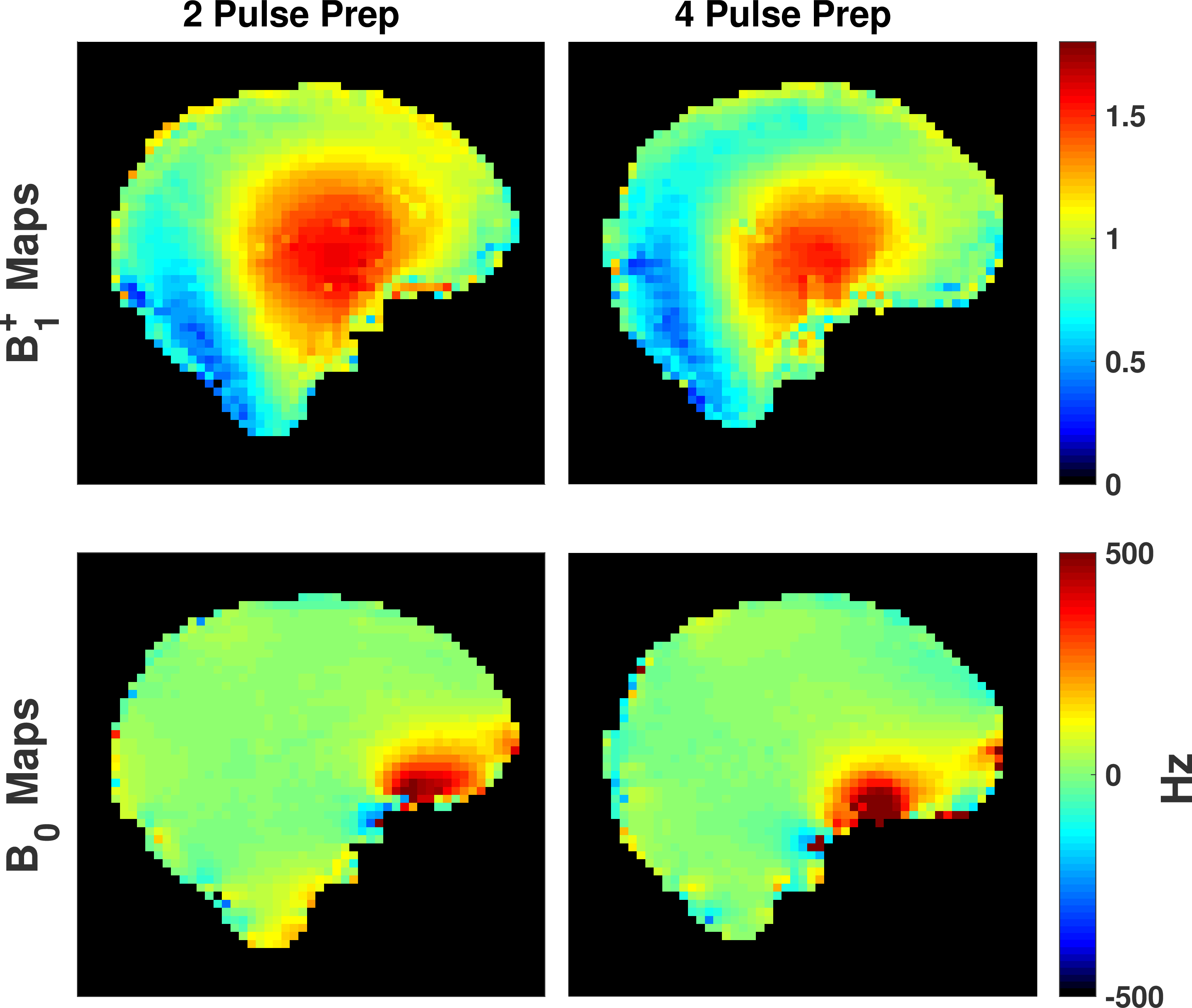


Fig S1 – B_1_^+^ maps (top row) and B_0_ maps (bottom row) used to produce the predictions in Figure 4. Data are from two separate subjects: the subject whose FLAIR imaging was performed with the 2-refocussing pulse MP is on the left and the 4-refocussing pulse MP subject is on the right.


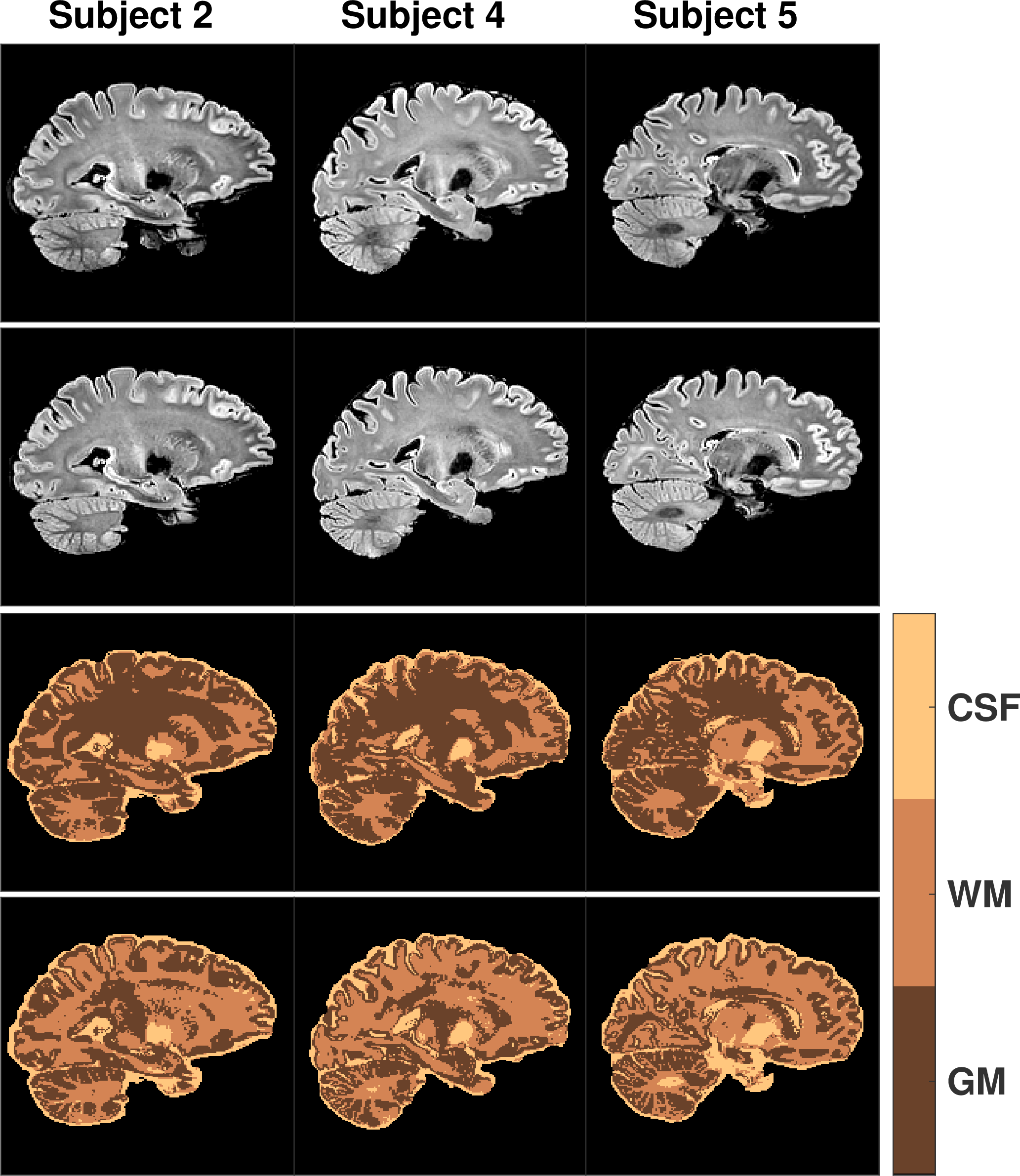


Fig S2 – Bias corrected and segmented images shown as in Figure 9 for the remaining three subjects.


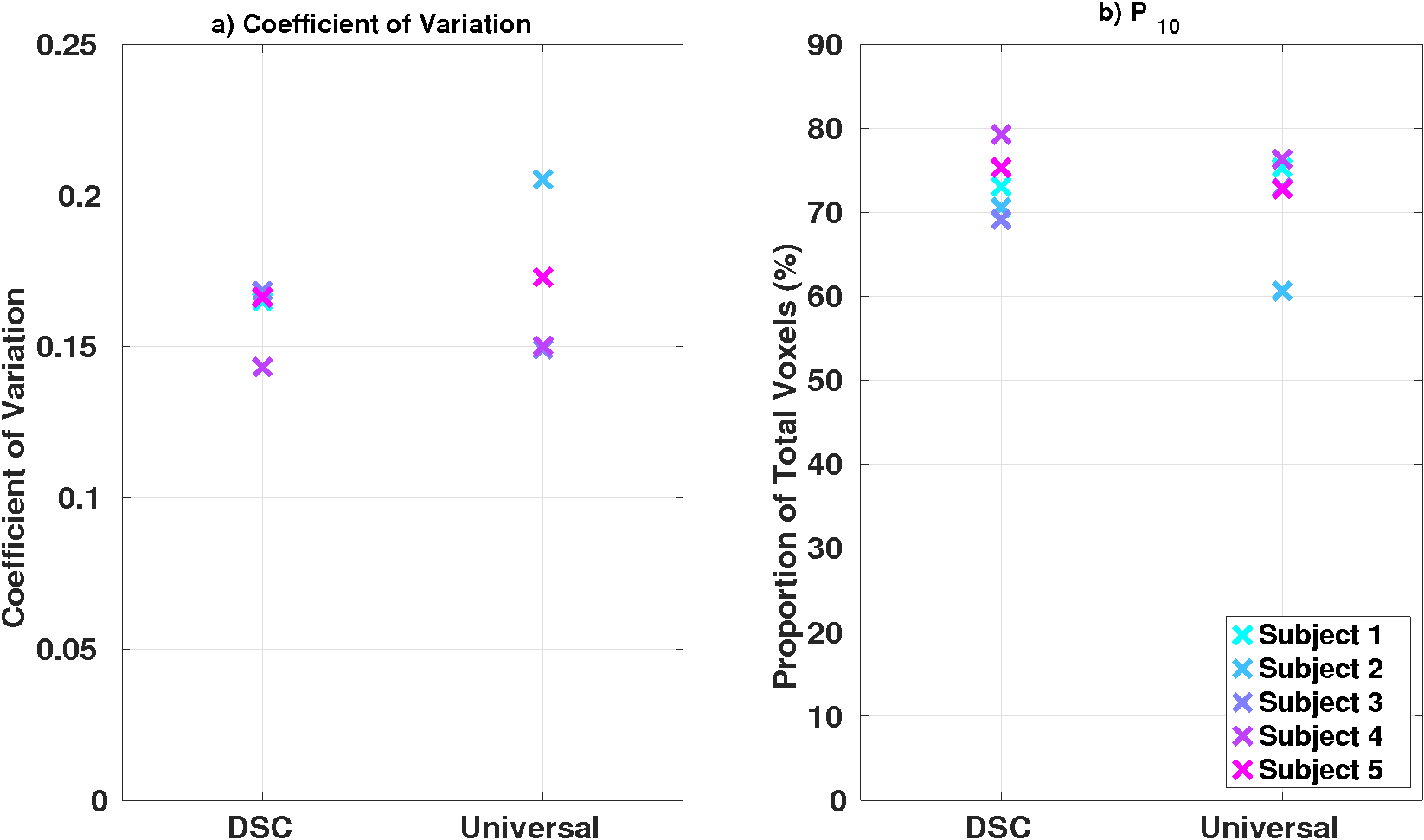


Fig S3 – **a)** Coefficient of variation in simulated signal for the central k-space echo over the whole brain shown for individual DSC (“DSC”) and universal DSC (“universal”) solutions. **b)** The P_10_ value is shown for the same datasets. The universal solutions lead to a larger spread in these metrics than the individually optimised solutions, however all are lower than the quadrature mode and static shimming cases (Fig. 7).


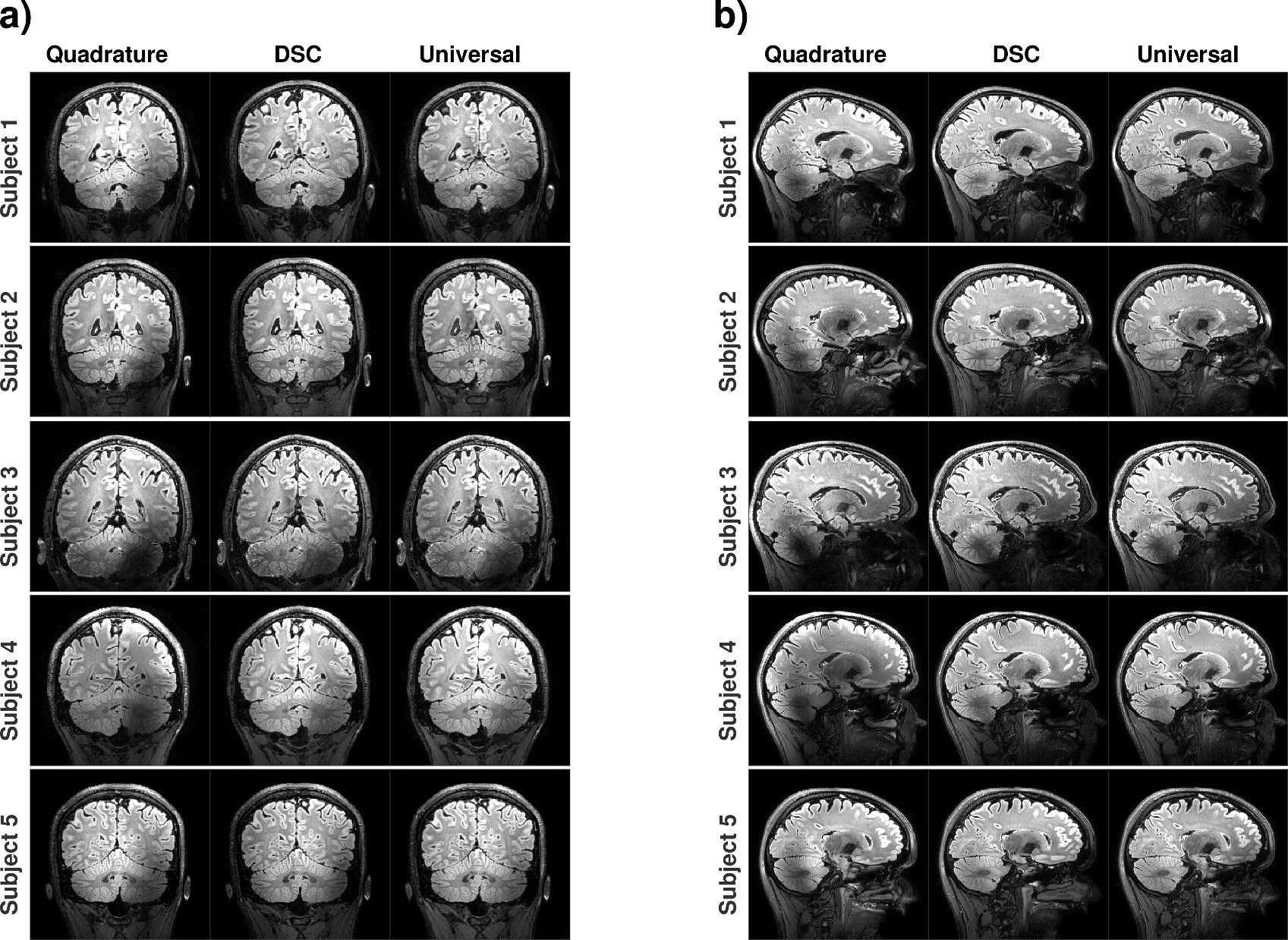


Fig S4 – Example image slices through the cerebellum in **a)** coronal view and **b)** sagittal view for all 5 imaged subjects for quadrature, individual DSC (“DSC”) and universal DSC (“universal”) solutions.
